# Cognitive Bias in the COVID-19 Pandemic

**DOI:** 10.7759/cureus.9019

**Published:** 2020-07-06

**Authors:** Christina N DiMaria, Byeori Lee, Robert Fischer, Glenn Eiger

**Affiliations:** 1 Internal Medicine, Einstein Medical Center, Philadelphia, USA; 2 Infectious Disease, Einstein Medical Center, Philadelphia, USA

**Keywords:** cognitive bias, covid, heuristics

## Abstract

Cognitive bias plays a significant role in medical errors. In the pandemic of corona virus disease-19 (COVID-19), recognizing and creating strategies to minimize these biases is crucial to optimize medical care for our patients. In this article we present a case of a 68-year-old male with decreased appetite, subjective fears, dry cough, and confusion. The report illustrates the concept of cognitive bias during a pandemic and discusses strategies to ameliorate them.

## Introduction

Cognitive bias includes a variety of unconscious influences, heuristics, and behaviors which aid our decision making [1]. These shortcuts can be helpful in clinical decision making, but they can also lead to medical errors. A study by Graber et al. found that 74% of diagnostic errors in internal medicine practice are related to cognitive factors [2]. Recognizing and creating strategies to minimize these biases is crucial to optimize medical care for our patients. We describe a case of a 68-year-old male with decreased appetite, subjective fears, dry cough, and confusion presenting during the corona virus disease-19 (COVID-19) pandemic. To our knowledge there are no case reports or studies focusing on the role of cognitive biases during a pandemic or strategies to overcome them.

## Case presentation

A 68-year-old male presented with decreased appetite, fatigue, and confusion for four days. His wife reported worsening disorientation and drowsiness for one day prompting her to bring him to the hospital. Other symptoms included subjective fever and dry cough. He lived at home with his wife and grandchildren and was independent in activities of daily living at baseline. He had hypertension and hyperlipidemia. The patient had no recent travel or sick contacts. His medications included spironolactone, hydrochlorothiazide, losartan, and nifedipine. He had no smoking, alcohol, or drug use history.

Vital signs on admission included blood pressure 135/77 mmHg, oxygen saturation of 97% on room air, heart rate 80 bpm, and temperature 39 degrees Celsius. He had no neck stiffness, no focal neurological deficits, and was alert and oriented to his name. The rest of the physical findings were unremarkable. Chest X-ray showed no acute disease. Noncontrast cranial CT was negative. Serum sodium was 140 mEq/L, potassium 3.8 mEq/L, creatinine 2.3 mg/dL, WBC 10,000 per microliter with a neutrophil predominance of 80%. A nasal swab was negative for influenza and respiratory syncytial virus (RSV) by polymerase chain reaction (PCR). A nasal swab for COVID-19 PCR was obtained and the patient was admitted to the COVID general medicine floor for “COVID rule out.”

While awaiting the COVID test result, the patient was followed clinically. Minimal additional testing was performed. His mental status continued to wax and wane for the next six days, with delirium worsening when his fever was high. No specialty services were consulted. Eventually, COVID-19 testing returned positive and he was treated with symptomatic management.

## Discussion

This case brings into question how we approach medical decision making during the COVID-19 pandemic. Although our patient was eventually diagnosed with COVID-19, there were alternative diagnoses that should have been considered including but not limited to a central nervous system (CNS) infection or vasculitis, seizures, hypercapnia, HIV, or uncontrolled hyperthyroidism.

The primary bias illustrated by our case is premature closure. Premature closure is a cognitive error in which the physician fails to consider reasonable alternatives after an initial diagnosis is suspected [3]. Given the increasing prevalence of COVID-19, it is hard not to focus on this diagnosis when evaluating a patient. After the patient was admitted to the medicine floor, minimal further testing was done as it was thought “prematurely” that the patient had COVID-19. Another cognitive bias demonstrated by this case is availability bias -- the mental shortcut that relies on likelihood or frequency of a disease to construct a differential diagnosis [4]. Currently there are 2,027,521 confirmed cases of COVID-19 in the United States [5]. The likelihood of our patient having COVID-19 is high, yet other diseases have not vanished, and other diagnoses are still possible.

On review of the case, the presenting symptoms were vague, yet we focused on dry cough and fever to lead us to a diagnosis of COVID-19. Other symptoms and other physical findings were overlooked. This is an example of anchoring bias.

The final bias observed in our case is framing bias, the influence of how information is presented on medical decision making [6]. This bias occurs at many levels; admission from the ED, medical rounds presentations, handoffs, and signouts. In this case, the general medicine team was called for an admission for “COVID rule out.” And this was also the term used for handoffs and signouts. We believe this wording may oversimplify the patient’s disease state and focus only on COVID-19.

Yes, in the end, the patient was found to have COVID-19, but what if after six days the test came back negative? Would we have then recognized the biases that affected our medical decision making? Could we have provided better and safer medical care? 

Recognition and strategies to prevent cognitive biases, especially during a pandemic, are crucial to optimize medical care for our patients. It would facilitate more efficient diagnosis of non-COVID-19 diseases which would allow for earlier discharges and free up hospital beds. For this reason, we suggest some guiding principles to prevent cognitive bias errors during the COVID-19 pandemic.

1. Three alternative diagnoses in addition to COVID-19

Three alternative diagnoses should be routinely sought for patients with suspected COVID-19 infection. After making sure the patient is screened appropriately for infection control and placed on the proper isolation, it is important to think about common pre-COVID-19 diseases. In addition, COVID-19 positivity does not rule out other diseases. Our patients are complicated and can have multiple diagnoses. Common things will always be common, and we are learning that this is still the case during a pandemic.

2. Diagnostic Time Out

After brainstorming differential diagnoses, take a time-out as a team. Ask what else besides COVID-19 can this be? As a team, review the patient’s initial symptoms, vital signs, labs and synthesize this with new data or findings found while the patient has been on the floor. During this time out consider how likely COVID-19 is for the patient and if potentially a positive test could be a false positive.

3. Include Specialists

We, physicians, are a team in this fight against COVID-19. Resources on many levels are stretched thin. For this reason, we think it would be beneficial to involve specialists. Even if they are working via telemedicine, discussing the case with experienced specialists may provide further insight.

## Conclusions

Although cognitive biases are known contributors to cognitive errors, we still are not sure of all the ways they play into medical decision making during this COVID-19 pandemic. By dissecting this case we feel we were able to illustrate the concept of cognitive bias, how it influences our medical care, and suggest a few strategies to overcome them.
